# COVID-19 Infections in Adults with Congenital Heart Disease—A Prospective Single-Center Study in an Outpatient Setting

**DOI:** 10.3390/jcm11206105

**Published:** 2022-10-17

**Authors:** Nora Langes, Christian Meierhofer, Nicole Nagdyman, Susanne J. Maurer, Felix Bourier, Martin Halle, Stefan Holdenrieder, Peter Ewert, Oktay Tutarel

**Affiliations:** 1 Department of Congenital Heart Disease and Paediatric Cardiology, German Heart Centre Munich, TUM School of Medicine, Technical University of Munich, 80636 Munich, Germany; 2 Department of Electrophysiology, German Heart Centre Munich, TUM School of Medicine, Technical University of Munich, 80636 Munich, Germany; 3 Department of Prevention and Sports Medicine, University Hospital Klinikum Rechts der Isar, Technical University of Munich, 81675 Munich, Germany; 4 DZHK (German Centre for Cardiovascular Research), Partner Site Munich Heart Alliance, 80992 Munich, Germany; 5 Institute of Laboratory Medicine, German Heart Centre Munich, TUM School of Medicine, Technical University of Munich, 80636 Munich, Germany

**Keywords:** adult congenital heart disease, COVID-19, cardiac MRI

## Abstract

Background: COVID-19 might pose a risk for adults with congenital heart disease (ACHD). However, data regarding the rate of infection as well as myocardial involvement in ACHD patients are currently lacking. Methods: During the study period from January to June 2021, all consecutive outpatients from our ACHD clinic were eligible to participate. Clinical data were collected. An antibody test for COVID-19 was performed in all patients. Cardiovascular magnetic resonance imaging (CMR) was offered to those with a positive antibody test. Results: Overall, 420 patients (44.8% female, mean age 36.4 ± 11.6 years) participated. Congenital heart defect (CHD) complexity was simple in 96 (22.9%), moderate in 186 (44.3%), complex in 117 (27.9%), and miscellaneous in 21 (5.0%) patients. Altogether, 28 (6.7%) patients had a positive antibody test. Out of these, 14 had an asymptomatic course. The others had mainly mild symptoms and were managed as outpatients. Furthermore, 11 patients (39.3%) had even not been aware of their infection. Fourteen patients underwent a CMR without signs of myocardial involvement in any of them. Conclusions: We observed a number of undetected cases of COVID-19 infections in our ACHD population. Reassuringly, in all cases, the infection had a mild clinical course.

## 1. Introduction

The number of adults with congenital heart disease (ACHD) is increasing [1]. Currently, it is estimated that adults account for two-thirds of patients with severe and other forms of congenital heart disease (CHD) in the general population [2]. These ACHD patients have to deal with residua and sequelae related to their CHD as well as acquired comorbidities, especially as they are aging [3]. These comorbidities are not just innocent bystanders but can determine the outcome of ACHD patients [4,5,6,7]. This combination of residua and sequela of CHD, as well as the prevalence of comorbidities, led to the assumption that ACHD patients could be an especially vulnerable population regarding an unfavorable outcome of a Coronavirus disease 2019 (COVID-19) infection caused by the severe acute respiratory syndrome coronavirus 2 (SARS-CoV-2) [8,9]. An international, multi-center study reported the outcome of 1044 ACHD patients with COVID-19 [10]. Several risk factors for a fatal outcome were identified including male sex, diabetes, cyanosis, pulmonary hypertension, and a worse physiological stage [10]. The authors concluded that “COVID-19 mortality in adults with CHD is commensurate with the general population” [10]. The majority of patients (94%) in this study had symptoms and were referred to specialized centers. However, many patients with COVID-19 remain asymptomatic, and the true rate of infection is unknown. This could also not be resolved by the large multicenter cohort data as the size of the population from which the cases were derived was missing [10]. A European study included 105 patients, which actively reported to their centers or were hospitalized for COVID-19 at the participating centers [11]. The presence of at least two comorbidities, overweight as well as cyanotic heart disease were identified as predictors for a complicated clinical course. However, the authors could not provide data regarding the prevalence and disease course of COVID-19 among their whole ACHD population because only patients with symptoms had been included. Hence, asymptomatic cases were also missed [11].

Furthermore, cardiac involvement in otherwise healthy non-cardiac patients recovering from COVID-19 has been described [12,13]. However, both studies in ACHD patients did not address the myocardial involvement question. Hence, it is not known if residua of a cardiac involvement of the COVID-19 infection are present.

Therefore, the main aim of our study was to report the rate of COVID-19 infections in unselected ACHD patients in an outpatient setting from a tertiary center. A secondary aim was to assess if there was myocardial involvement after recovery from a COVID-19 infection.

## 2. Materials and Methods

In this prospective single-center study, all consecutive ACHD outpatients (>18 years) during the period January 2021–June 2021 were eligible to participate. Exclusion criteria were an age below 18 years and the absence of a CHD. 

After providing written informed consent, data, including demographics and clinical information (including a previous COVID-19 infection), were collected from the patients directly and from medical records. All patients underwent the standard protocol for a visit to our outpatient clinic, including a physical examination, an ECG, and an echocardiogram. 

The complexity of CHD was classified according to the Bethesda classification, which divides CHD into three groups: complex, moderate, and simple lesions [14]. Those who did not fit into these groups were classified as miscellaneous (e.g., patients with Marfan syndrome or other aortopathies, anomalous left coronary artery from the pulmonary artery, etc.). Subjective exercise capacity was graded according to the New York Heart Association (NYHA) classification. Cyanosis was defined as a resting oxygen saturation below 90%. Lung disease included any form of it (i.e., asthma, chronic obstructive lung disease, emphysema, etc.). Diabetes included both insulin-dependent and non-insulin-dependent cases. Endocrinological diseases included thyroid disorders. 

### 2.1. Laboratory Analysis

Blood was drawn for routine laboratory analyses in all patients. These included full blood count measured on the Sysmex XN 2000 (Sysmex Diagnostics, Norderstedt, Germany), electrolytes, creatinine, aspartate amino transferase (AST), bilirubin, and C-reactive protein (CRP) assessed on the Cobas C501 analyzer (Roche Diagnostics, Mannheim, Germany) as well as high sensitive troponin T, NT-pro-brain-natriuretic peptide (NT-proBNP), and anti-SARS-CoV-2 antibodies measured on the Cobas E411 analyzer (Roche Diagnostics, Mannheim, Germany). The Elecsys anti-SARS-CoV-2 antibody test is an immunoassay based on electrochemoluminescence technology (ECLIA) detecting qualitatively antibodies directed against the nucleocapsid antigen of SARS-CoV-2, thereby identifying persons who were infected with the SARS-CoV-2 virus in the past. All analyses were performed in the certified central laboratory of the German Heart Center Munich according to strict quality control guidelines of the German Federal Medical Council (Rili-BÄK).

### 2.2. Positive Rate of Antibodies

The positive rate for COVID-19 antibodies in our study was compared with the rate in the general German population during the same time period. These are publicly available at the Website of the Robert Koch Institute, the central governmental institution in the prevention and combating of infectious diseases in Germany (https://www.rki.de/DE/Content/InfAZ/N/Neuartiges_Coronavirus/Testzahl.html, accessed 19 September 2021).

### 2.3. International Physical Activity Questionnaire (IPAQ)

The German version of the IPAQ short form—an open-access physical activity questionnaire—was used to assess physical activity levels within the last seven days. Three questions on activities (vigorous, moderate, or walking) are used to assess three levels (categories) of physical activity: low, moderate, and high. It has been shown that the results of the IPAQ correlate with exercise capacity [15]. 

### 2.4. Cardiovascular Magnetic Resonance 

Cardiovascular magnetic resonance imaging (CMR) was offered to all patients who tested positive for COVID-19 antibodies. CMR was performed on a 1.5 Tesla MR scanner (Magnetom Avanto, Siemens Healthineers, Erlangen, Germany) with a standard 12-element cardiac phased array coil and with patients in a supine position, both before and after intravenous application of an extracellular MR contrast agent (gadopentetat-dimeglumine 0.15 mmol/kg). Cine images (balanced steady-state free precession, TR 45 ms, TE 1.3 ms, voxel size 1.8 × 1.8 × 8.0 mm, 25 phases) were acquired in short axis orientation. T2 turbo spin echo sequences and T2 mapping were acquired in all patients. T1 paramagnetic mapping using modified Look-Locker inversion recovery imaging (MOLLI) was performed using a pulse sequence before and 10 min after administration of the contrast agent. Late gadolinium enhancement (LGE) was acquired using a T1-weighted phase-sensitive inversion recovery sequence (PSIR) 15 min after intravenous administration of the contrast agent. Analysis of ventricular volumes, paramagnetic mapping, and LGE was performed by using CVI42^®^ (Circle Cardiovascular Imaging Inc., Calgary, AB, Canada). A myocardial involvement was defined according to current recommendations: myocardial edema by T2 mapping or T2-weighted imaging, myocardial injury by T1 mapping, myocardial injury by late gadolinium enhancement, pericardial changes, new ventricular function abnormalities, or other new cardiac structural abnormalities [16]. 

### 2.5. Statistical Analyses 

Statistical analyses were performed using SPSS version 25 (IBM Corp. Armonk, NY, USA) and MedCalc Statistical Software version 19.2.1 (MedCalc Software Ltd. Ostend, Belgium). Continuous variables are presented as mean ± standard deviation or median (interquartile range) depending on data distribution, whereas categorical variables are presented as numbers (percentage). Comparisons between groups were made using the Mann–Whitney U test, Student’s *t*-test, Fisher’s exact test, or Chi-square test as appropriate. All tests were performed two-sided and for all analyses a *p*-value < 0.05 was considered statistically significant.

## 3. Results

**Figure 1:**
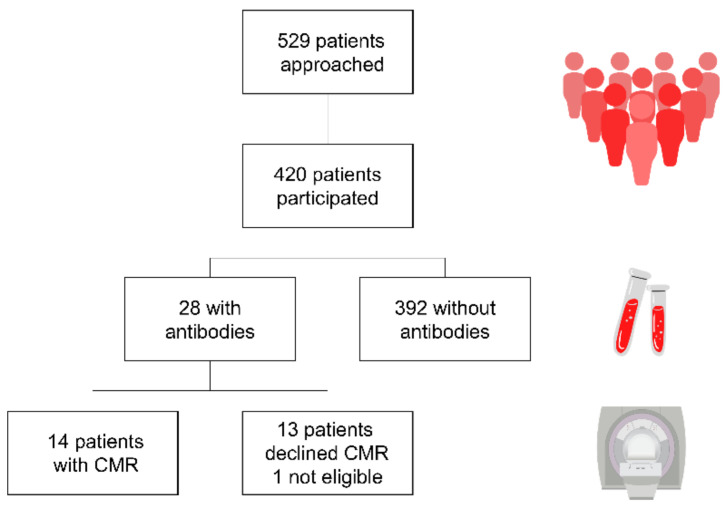
Study population.

Altogether, 549 patients were approached for the study and out of these, 420 patients (44.8% female, mean age 36.4 ± 11.6 years) agreed to participate (Figure 1).

The CHD was a simple defect in 96 (22.9%), a defect of moderate complexity in 186 (44.3%), a complex defect in 117 (27.9%), and a miscellaneous defect in 21 (5.0%) patients. Out of the 420 patients, 300 (71.4%) were in NYHA class I, 80 (19.0%) in class II, and 21 (5.0%) in class III. In 19 patients (4.5%), NYHA class was not documented. Cyanosis was present in 22 patients (5.2%). More details are provided in Table 1 and Table A1, Appendix A. 

### 3.1. COVID-19

**Figure 2:**
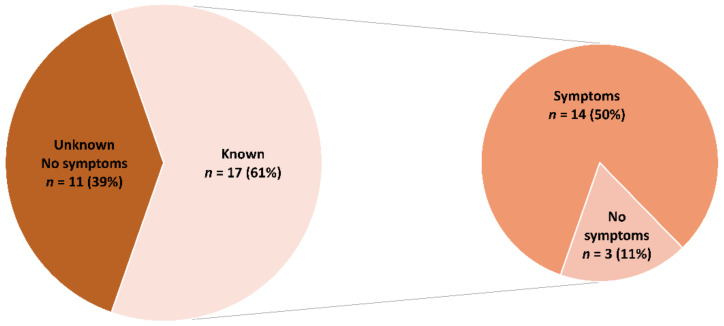
Knowledge and symptoms of a COVID-19 infection in patients with a positive antibody test.

Altogether, 28 (6.7%) patients (CHD complexity: simple *n* = 5, moderate *n* = 14, severe *n* = 7, miscellaneous *n* = 2) had antibodies for COVID-19. Out of these, 17 patients were aware of their previous COVID-19 infection, while 11 patients (39.3%) were not and also had no symptoms (Figure 2).

Fourteen of the 28 patients had an asymptomatic infection. Symptoms were mild in the remaining patients, and all were treated as outpatients. Hospitalization was not necessary for any of the patients. When comparing the groups with a positive antibody test and those with a negative test, no significant difference was found for a variety of clinical variables (Table 1). 

### 3.2. Positivity Rate

In our cohort, 6.7% of patients had COVID-19 antibodies. For the whole German population, the positivity rate during the study period was 8.1%.

### 3.3. Immunization/Vaccination

Out of the 420 patients, 66 (15.7%) had received their first dose of a COVID-19 vaccine, while 42 (10.0%) had received their second dose. 

### 3.4. Laboratory

Values for troponin T (5.8 ± 3.7 vs. 6.4 ± 4.9 ng/L, *p* = 0.574) as well as for NT-proBNP (171 ± 181 vs. 265 ± 470 ng/L, *p* = 0.391) did not significantly differ between patients with antibodies and those without. There was also no significant difference for other laboratory values (Table 2).

### 3.5. International Physical Activity Questionnaire (IPAQ)

A low physical activity level was reported by 77 patients (18.3%), a moderate level by 139 (33.1%), and a high level by 192 (45.7%). Twelve patients did not complete the questionnaire.

### 3.6. CMR 

Fourteen of the 28 patients with COVID-19 antibodies consented to a CMR. Reasons cited for not participating in the CMR study were: not enough time, “feeling well”, and claustrophobia. One patient had a pacemaker that was not MRI-compatible. In the 14 patients who participated, myocardial involvement, i.e., signs of active or healed myocarditis could not be detected. 

## 4. Discussion

In this study, we found an infection rate for COVID-19 in unselected ACHD patients attending the outpatient clinic of a tertiary center of 6.7%. While 50% of infected patients were asymptomatic, around 40% of infected patients were unaware of their infection. In addition, patients after a COVID-19 infection who underwent a CMR showed no signs of myocardial involvement.

We observed a mild course of COVID-19 in all infected ACHD patients with 50% of patients even being asymptomatic. This is a reassuring finding and resembles observations from the general population as well as other groups deemed at higher risk for a worse outcome of a COVID-19 infection [17]. For the risk stratification of ACHD patients, it was noted that anatomic complexity alone is insufficient and that physiological aspects and patient status are more important [9]. In a single-center study from New York City, a worse clinical status and the presence of a genetic syndrome were predictors for a more severe disease course of a COVID-19 infection [18]. In a European multicenter study, predictors of a worse clinical course were age, body mass index, and the presence of cyanotic CHD [11]. In the largest study so far by Broberg et al., risk factors for mortality included male sex, diabetes, cyanosis, pulmonary hypertension, renal insufficiency, and worse physiological stage [10]. This might explain the mild course in our ACHD population. While around 72% of the patients had a CHD of moderate or severe complexity, the vast majority were in a good clinical status, exemplified by the fact that 91% were in NYHA class I and II. However, we found no difference between patients with COVID-19 antibodies and those without antibodies regarding their body mass index, comorbidities, or the presence of cyanosis. Furthermore, older age has been identified as a risk factor for a worse outcome in COVID-19 [19]. However, our cohort was quite young, with a mean age of 36 years, and this might add an additional layer of protection. 

In the current study, all patients were seen in an outpatient setting. A recent publication using administrative data reported a worse clinical course in ACHD patients hospitalized for COVID-19 compared to patients without a CHD, albeit with the same mortality [20]. Therefore, our study could be biased by not including hospitalized patients. However, during the study period, there was no ACHD inpatient admitted for COVID-19 in our large tertiary ACHD center. Furthermore, while we cannot rule out that a patient was treated in an inpatient setting in a different hospital, we are generally contacted for any of our ACHD patients who are admitted to an outside hospital for an acute disease. However, we might have missed patients with an ongoing COVID-19 infection, who were not hospitalized, but decided not to come to their outpatient appointment due to their infection. 

The positivity rate for COVID-19 antibodies in our ACHD patients was comparable to that of the general population in Germany for the study period. This is an interesting finding considering that early on in the pandemic, some European centers had—for selected ACHD patients—not only recommended social distancing and face masks but also shielding, i.e., patients were advised to stay home at all times, and also should keep distance from other household members [21]. Such isolation would certainly add to the already considerable emotional toll the pandemic had on ACHD patients as well as the general population [22,23]. However, our results suggest that the risk behavior regarding protection from a COVID-19 infection was not different between ACHD patients and the general population. While a study from the UK reported a significant decrease in the physical activity level in ACHD patients mainly due to fear of COVID-19 [24], our study cohort showed similar levels of physical activity assessed by the IPAQ questionnaire compared with a pre-pandemic study from our center [15]. 

In patients recovered from COVID-19, cardiac involvement with ongoing inflammation on CMR has been described in patients with and without cardiac symptoms [12,13,25]. A recent study confirmed these findings in patients recovering from the Delta variant of COVID-19 [26]. In our study, none of the patients who underwent CMR showed cardiac involvement. This could be attributed to the fact that our ACHD patients with COVID-19 infection were younger and had only mild or no symptoms. In accordance, a study of children who recently recovered from mildly symptomatic COVID-19 infections showed no evidence of myocardial inflammation, fibrosis, or functional cardiac impairment on CMR [27]. Furthermore, the rate in young athletes was also lower than those previously reported in an older population [28]. Hence, these results are reassuring, however, do not rule out cardiac involvement in ACHD patients with more severe symptoms of COVID-19 requiring hospitalization or treatment in the intensive care unit. 

Interestingly, only 15.7% of our patients had received their first dose of a COVID-19 vaccine, while 10.0% had received their second dose. While these numbers may seem low, they have to be viewed in the context of the availability of vaccines. During the study period, the roll-out of vaccines just had started, and at that time, not enough vaccinations to meet demands were available in Germany. The situation improved in the second half of 2021. Therefore, we assume that the current number of ACHD patients with completed vaccinations is much higher. 

A limitation of this study is that, during the study period, the most prevalent variants in Germany were Alpha and Delta. Therefore, we do not know if the data are applicable to the current situation with different variants. However, considering the probable more benign disease course in current variants and the uptake of COVID-19 vaccinations, we would not expect significantly different results now. A further limitation is that only 14 out of 28 patients with positive COVID-19 antibodies underwent a CMR. One patient was not eligible due to the implanted pacemaker system, the other 13 patients refused the CMR mainly due to “not feeling ill”, time constraints, and claustrophobia. Since these patients also had only mild or no symptoms during their COVID-19 infection; we do not believe that the CMR results would be different.

## 5. Conclusions

In conclusion, 6.7% of ACHD outpatients in a tertiary ACHD center had COVID-19 antibodies. Half of these were asymptomatic, while around 40% were unaware of the prior infection. None of the patients who had a CMR showed signs of myocardial involvement. Therefore, there is a number of undetected cases of COVID-19 infections in ACHD patients with a benign clinical course.

## Figures and Tables

**Table 1:** Baseline characteristics.

	All, *n* (%)	COVID-19 Antibodies Neg., *n* (%)	COVID-19 Antibodies Pos., *n* (%)	*p*
N	420	392	28	
Mean age in years	36.4 ± 11.6	36.6 ± 11.6	33.0 ± 10.0	0.103
Female	188 (44.8)	174 (44.8)	14 (50.0)	0.564
Body mass index (kg/m^2^)		27.3 ± 50.7	25.0 ± 4.0	0.846
<25	236 (56.2)	220 (59.1)	16 (57.1)	
25–30	120 (28.6)	112 (28.6)	8 (28.6)	
>30	44 (10.5)	40 (10.2)	4 (14.3)	
missing	20 (4.8)	20 (5.1)	0	
Complexity				0.822
Simple	96 (22.9)	91 (23.2)	5 (17.9)	
Moderate	186 (44.3)	172 (43.9)	14 (50.0)	
Severe	117 (27.9)	110 (28.1)	7 (25.0)	
Miscellaneous	21 (5.0)	19 (4.8)	2 (7.1)	
Cyanosis	22 (5.2)	20 (5.1)	2 (7.1)	0.640
History of arrhythmias	49 (11.7)	46 (11.7)	3 (10.7)	0.871
Heart rhythm at presentation				
Sinus rhythm	371 (88.3)	344 (87.8)	27 (96.4)	0.23
Pacemaker	34 (8.1)	33 (8.4)	1 (3.6)	0.72
Atrial fibrillation	2 (0.5)	2 (0.5)	0 (0)	1.00
Other	3 (0.7)	3 (0.8)	0 (0)	1.00
Not documented	10 (2.4)	10 (2.6)	0 (0)	1.00
NYHA class				0.444
I	300 (71.4)	279 (71.2)	21 (75.0)	
II	80 (19.0)	74 (18.9)	6 (21.4)	
III	21 (5.0)	21 (5.4)	0 (0)	
Not documented	19 (4.5)	18 (4.6)	1 (3.6)	
Vaccination (COVID-19)				0.358
First dose	66 (15.7)	62 (15.8)	4 (14.3)	
Second dose	42 (10.0)	40 (10.2)	2 (7.1)	
None	312 (74.3)	290 (74.0)	22 (78.6)	
Genetic syndrome	39 (9.3)	37 (9.2)	2 (7.1)	0.686
Comorbidities				
CVA	36 (8.6)	34 (8.7)	2 (7.1)	0.780
Arterial hypertension	52 (12.4)	49 (12.5)	3 (10.7)	0.782
Lung diseases	41 (9.8)	39 (9.9)	2 (7.1)	0.629
Diabetes	12 (2.9)	11 (2.8)	1 (3.6)	0.568
Liver diseases	40 (9.5)	38 (9.7)	2 (7.1)	0.657
Renal diseases	31 (7.4)	31 (7.9)	0 (0)	0.122
Endocrinologic diseases	61 (14.5)	61 (15.6)	0 (0)	0.02
Gastrointestinal disorders	25 (6.0)	22 (5.6)	3 (10.7)	0.270
Rheumatological disorders	8 (1.9)	8 (2.0)	0 (0)	0.445

NYHA: New York Heart Association; CVA: cerebrovascular accident; Values for body mass index were missing in 20 patients.

**Table 2:** Comparison of different laboratory values between COVID-19 antibodies positive and negative patients.

	COVID-19 Antibodies Neg. (*n* = 392)	COVID-19 Antibodies Pos. (*n* = 28)	*p*
Haemoglobin, g/dL	14.7 ± 1.9	15.0 ± 2.0	0.592
Haematocrit, %	43.4 ± 6	44.0 ± 6	0.618
Thrombocytes, /µL	229,690 ± 57,533	222,070 ± 74,510	0.316
Leukocytes, /µL	6594 ± 1867	6505 ± 1768	0.954
Potassium, mmol/L	4.0 ± 0.3	4.1 ± 0.3	0.859
Sodium, mmol/L	138 ± 2	138 ± 2	0.509
Creatinine, mg/dL	0.89 ± 0.36	0.86 ± 0.13	0.833
GFR, mL/min	99 ± 22	106 ± 17	0.169
Troponin T, ng/L	6.4 ± 4.9	5.8 ± 3.7	0.574
AST, U/L	25.4 ± 16.8	25.5 ± 11.0	0.691
Bilirubin, mg/dL	0.74 ± 0.60	0.86 ± 0.87	0.825
CRP, mg/L	2.3 ± 5.1	1.8 ± 1.8	0.316
NT-proBNP, ng/L	265 ± 470	171 ± 181	0.391

CRP: C-reactive protein; GFR: glomerular filtration rate; AST: aspartate aminotransferase.

## Data Availability

The data underlying this article cannot be shared publicly due to data privacy reasons and the according German regulations.

## Appendix A

**Table A1:** Type of congenital heart defect, *n* (%).

Main Congenital Heart Defect	All (*n* = 420)	COVID-19 Antibodies Neg. (*n* = 392)	COVID-19 Antibodies Pos. (*n* = 28)
Atrial septal defect	31 (7.4)	30 (7.7)	1 (3.6)
Ventricular septal defect	38 (9.0)	34 (8.7)	4 (14.3)
Atrioventricular septal defect	19 (4.5)	19 (4.8)	0
Patent ductus arteriosus	3 (0.7)	3 (0.8)	0
Isolated valve disease excluding PS	57 (13.6)	53 (13.5)	4 (14.3)
Pulmonary valve stenosis	20 (4.8)	20 (5.1)	0
Sub- or supravalvular aortic stenosis	9 (2.1)	9 (2.3)	0
Coarctation of the aorta	43 (10.2)	41 (10.5)	2 (7.1)
Tetralogy of Fallot	51 (12.1)	47 (12.0)	4 (14.3)
Ebstein’s anomaly	18 (4.3)	14 (3.6)	4 (14.3)
Transposition of the great arteries	49 (11.7)	45 (11.5)	4 (14.3)
Pulmonary atresia	14 (3.3)	13 (3.3)	1 (3.6)
Double outlet right ventricle	14 (3.3)	13 (3.3)	1 (3.6)
Truncus arteriosus	3 (0.7)	3 (0.8)	0
Anomalous pulmonary venous connection (partial/total)	6 (1.4)	6 (1.5)	0
Single ventricle physiology	22 (5.2)	21 (5.4)	1 (3.6)
PAH/Eisenmenger syndrome	2 (0.5)	2 (0.5)	0
Other *	21 (5.0)	19 (4.8)	2 (7.1)

PS: pulmonary valve stenosis; PAH: pulmonary arterial hypertension; *: including Marfan syndrome, aortopathies, etc.
